# Decoding the role of coiled-coil motifs in human prion-like proteins

**DOI:** 10.1080/19336896.2021.1961569

**Published:** 2021-08-24

**Authors:** Molood Behbahanipour, Javier García-Pardo, Salvador Ventura

**Affiliations:** Institut De Biotecnologia I De Biomedicina (Ibb) and Departament De Bioquímica I Biologia Molecular, Universitat Autónoma De Barcelona, Barcelona, Spain

**Keywords:** Prions, amyloids, prion-like domains, coiled-coils, protein aggregation

## Abstract

Prions are self-propagating proteins that cause fatal neurodegenerative diseases in humans. However, increasing evidence suggests that eukaryotic cells exploit prion conformational conversion for functional purposes. A recent study delineated a group of twenty prion-like proteins in humans, characterized by the presence of low-complexity glutamine-rich sequences with overlapping coiled-coil (CCs) motifs. This is the case of Mediator complex subunit 15 (MED15), which is overexpressed in a wide range of human cancers. Biophysical studies demonstrated that the prion-like domain (PrLD) of MED15 forms homodimers in solution, sustained by CCs interactions. Furthermore, the same coiled-coil (CC) region plays a crucial role in the PrLD structural transition to a transmissible β-sheet amyloid state. In this review, we discuss the role of CCs motifs and their contribution to amyloid transitions in human prion-like domains (PrLDs), while providing a comprehensive overview of six predicted human prion-like proteins involved in transcription, gene expression, or DNA damage response and associated with human disease, whose PrLDs contain or overlap with CCs sequences. Finally, we try to rationalize how these molecular signatures might relate to both their function and involvement in disease.

## INTRODUCTION

Prusiner and co-workers coined the term prion in 1982. They reported the purification of infectious particles from scrapie-infected hamster brains for the first time and demonstrated that they consisted of a specific type of pathogenic element that causes fatal neurodegenerative diseases in mammals and humans [1,2]. It has been assumed that the toxicity of prions relies on their intrinsic capacity to lose their native conformation (either in ordered or disordered regions) and acquire a β sheet-rich secondary structure with significant aggregation and self-propagating capacities. However, almost forty years after this seminal discovery, increasing evidence indicates that prion-like conformational conversion is not always pathogenic. On the contrary, it can be exploited for functional roles [3]. The best-characterized examples of non-pathogenic prions are those identified in yeast and filamentous fungi. Both yeast and fungal prions are multi-domain proteins with regions enriched in Asn (N) and/or Gln (Q) residues [2,4]. Typically, these long Q/N-rich sequences map into unstructured regions of the protein termed prion domains (PrDs). Upon a conformational switch to a self-perpetuating conformation, the prionic traits can be inherited during cell division in a non-Mendelian manner. This atypical mechanism of protein transmission provides increased tolerance to stress and facilitates adaptation to changing environments [5,6].

A less recognized feature of PrDs is the presence of protein sequence stretches with a propensity to form coiled-coils (CCs), often overlapping with the Q/N-rich or polyQ regions [3,7]. This super secondary structure has been associated with the formation of dimers, trimers, and higher-order oligomeric structures. Furthermore, it has been proposed that coiled-coil (CC) formation is an essential step preceding the physiological conformational switches to amyloid-like states in yeast prions [3,7].

Our group recently exploited different bioinformatics tools to systematically analyse the presence of prion-like domains (PrLDs) at the proteome level [2,3,8]. Using this approach, we identified a subset of human polypeptides that bear PrLDs with properties resembling those of yeast PrDs [3,8]. Subsequently, we explored whether these human domains contain regions with the propensity to fold into CCs using the COILS [9] and PARCOIL2 [10] algorithms. A total of 22 of these PrLDs were predicted to display sequences with a high propensity to fold into CCs, with 20 of them presenting polyQ tracts of different lengths. Interestingly enough, we observed that the majority of the identified proteins, if not all, participate in transcriptional regulation. This is the case of the mediator complex subunit 15 (MED15). MED15 is part of the mediator multi-protein complex that regulates enhancer-driven gene transcription [3] and, under normal conditions, is located in the nucleus [3]. Thus, MED15 knockdown has been linked to reduced growth and decreased transcriptional activation, while MED15 is highly overexpressed in different human cancers, including head and neck squamous cell carcinomas, hepatocellular carcinoma, breast cancer, renal cell carcinoma, and testicular germ cell tumours. Indeed, patients with MED15 over-expression in tumour tissues exhibit bad prognosis, significantly shorter survival times, and more aggressive phenotypes [11]. MED15 contains a disordered N-terminal low-complexity region composed of discontinuous polyQ tracts and a strong propensity to form CCs that map to its PrLD.

Recent work has focused on elucidating the role of the CC motif for structural transitions of initially soluble α-helical regions to β-sheet amyloid states. This has been particularly challenging to study in the past in the context of complete PrLDs, and previous reports dealt with only relatively short peptides derived from yeast prions (*i.e*., Ure2p PrD). Recently, Batlle and co-workers have experimentally demonstrated, for the first time, that is, in fact, the CC motif of MED15 PrLD that mediates the transition towards a β-sheet amyloid state [3]. They proposed that this behaviour may prevent the establishment of relevant protein-protein interactions in pathological situations. Alternatively, MED15 self-association might result in phase separation and the consequent formation of transcriptional hubs. A third possibility is that the PrLD of MED15 would establish pathological interactions with other Q-rich proteins, leading to the formation of cytoplasmatic insoluble aggregates. All in all, these recent results reinforce the hypothesis that aggregation of MED15 PrLD into amyloid fibrils involves a progressive conformational switch rather than a simple uncontrolled misfolding mechanism. It is feasible that the exact mechanism applies to the rest of the 19 identified polypeptides. Here we discuss this possibility and its potential implications for six of these predicted prion-like proteins associated with human disease.

## LOW COMPLEXITY SEQUENCES IN PRION-LIKE PROTEINS

Prion-like proteins are rich in low complexity sequences. Recent work has shown that binding of low complexity regions to their physiological partners is often accompanied by a local increase in the structuration of the binding region in a process known as ‘folding upon binding’. This binding and folding mechanism has been previously reported for different CCs, such as the GCN4 CC dimerization domain. This domain shows a two-state unfolding transition in which the binding appears coupled to folding [12]. Similarly, thermodynamic analyses with the CC oligomerization domain (SARAH) from serine/threonine mammalian sterile 20-like kinase (MST1) demonstrated that this domain is unstructured and folds upon binding to different partners [13,14]. However, other structural studies of the GCNA4 CC indicated that a pre-existing helical content promotes complex formation [14–16], which shows that segments of intrinsic helical propensity are important drivers of the interaction. As mentioned, a significant proportion of human prion-like proteins display sequences with a high propensity to fold into CCs that overlap with low complexity sequences. Thus, it is tempting to speculate that the presence of these preformed helical regions may speed up the binding to their partners. After this initial contact, further local folding may still take place within the complex.

## COILED COILS MEDIATE FUNCTIONAL INTERACTIONS AND AMYLOID FORMATION

**Figure 1.**
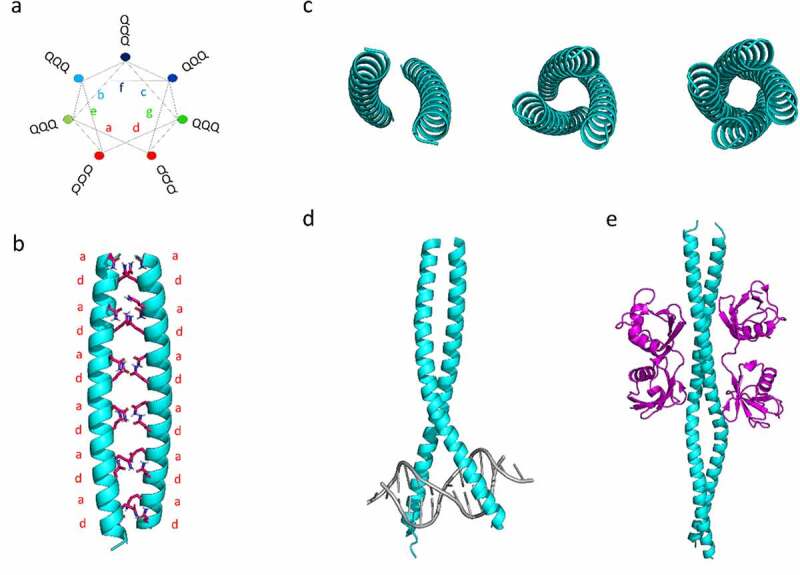
**Structural features of coiled-coils built up by Poly-Q repeats**. a) Helical wheel illustration for a regular heptad pattern composed built up by 21 Gln residues. The Gln residues are found in all seven (a-b-c-d-e-f-g) positions of the heptad repeat due to the particular chemical nature of this amino acid. b) Modelled structure of a canonical CC composed of 42 Gln residues. c) Structural representation of the oligomerization states driven by the supercoiling of the α-helices in a parallel orientation. d,e) Examples of functional protein-DNA and protein-protein interactions mediated by CCs. d) X-ray crystal structure of the leucine zipper Pap1 bZIP dimer bound to DNA. e) Crystal Structure of a diUb chain bound to the NEMO-UBAN domain. PDB codes are: 1GD2 and 2ZVN for d) and e), respectively

Sequences with Poly-Q repeat motifs are known to have a high propensity to form CCs. These sequences are frequent in yeast prions [4] and human proteins bearing PrLDs [3,7]. CC motifs were traditionally considered as molecular spacers between functional domains [17]. However, a growing body of evidence suggests that they frequently contain interaction regions and act as protein-protein interactors (PPI) and/or catalytic effectors [17]. CC forming proteins are widely distributed and have been shown to play a variety of biological roles [17,18], such as transcription regulation (*i.e*., leucine zippers) [19]; modulation of chromatin [20]; chromosome dynamics [21]; kinetochore assembly [22]; cell cycle progression [23]; organization of the centrosome [24]; vesicle transport [25], organelles structuration and activation of cell-signalling cascades [26,27], among other functions. Examples of these functions are depicted in Figure 1. CC motifs are particularly abundant in RNA-binding proteins (RBPs) that are localized to liquid-liquid phase-separated (LLPS) neuronal granules [18]. This phase transition has been shown essential for appropriate RNA trafficking during local protein synthesis, but it is also associated with misfolding and amyloid formation in various human neurological disorders [18].

The structure of CCs is now well understood, and this fold is ubiquitous in all domains of life (comprising up to 10% of all proteins in a given species) [25]. Naturally occurring CCs consist of at least two supercoiled α-helices. In their most prevalent form, they are built up from a heptad repeat *abcdefg*, in which the amino acids at positions *a* and *d* are mostly conserved hydrophobic residues (see Figure 1a). The resultant α-helices display a high amphipathic character, with both hydrophobic and polar faces. Yeast PrDs and human PrLDs possess sequence stretches in which Gln residues are abundant. Gln is both neutral and polar amino acid, with a long side chain that can be located on the surface of α-helices and at the theoretically hydrophobic a/d positions of the heptad repeats (see Figure 1a and Figure 1b). This dual behaviour allows for a variety of energetically favourable oligomerization states driven by the supercoiling of the α-helices (Figure 1c). Thus, it has been proposed that CC-mediated oligomerization of Q-rich proteins could drive to amyloid formation, given appropriate stimulus or induced by changes in the environmental conditions. In support of this hypothesis, Hartmann and co-workers reported that insertions of two or six residues strain the supercoil and lead to the local formation of β‐strands (also known as α‐β coiled‐coil) [28]. Additionally, experimental data indicate a possible direct structural conversion of CCs themselves to cross β-sheet amyloids. This structural shift was demonstrated in short peptides that form α-helical CCs when subjected to heating or pH changes [7], implying a structural shift from the intramolecular hydrogen-boding network that maintains CCs to the typical intermolecular-bonding landscape present in the β-sheets amyloids [29].

As discussed above, a recent study by our group supports this mechanism being behind the formation of highly ordered amyloids by the PrLDs of MED15. Other human proteins that may respond to the exact mechanism were discovered in the same study but could not be discussed in detail. In the following sections, we describe the cases of TATA-box-Binding Protein (TBP), cAMP-response element-protein-(CREB) binding protein (CBP), ataxin-1 (ATXN-1), ataxin-8 (ATXN-8), Lysine Methyltransferase 2D (KMT2D), and Forkhead box protein P2 (FOXP2), as representatives of this set of polypeptides.

## TATA-BOX-BINDING PROTEIN (TBP)

**Figure 2.**
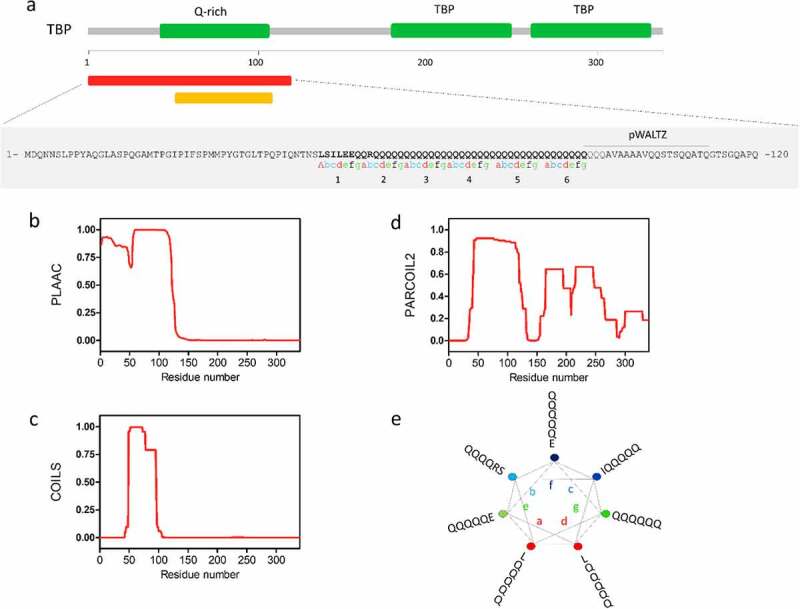
**TBP contains a Q-rich coiled-coil PrLD**. a) Linear representation of human TBP, showing the location of the N-t Q-rich sequence and the pair of C-t TATA-box-binding domains. b) The predictions for the prion-like domain (red) identified using PLAAC (30) predictor. CC per-residue probability of TPB calculated using c) COILS [9] and d) PARCOIL2 [10]. d) Wheel representation of the heptad repeats (a-b-c-d-e-f-g) of TBP. The sequence corresponding to the soft amyloid core (SAC) of TPB predicted by pWALTZ [45] is indicated

The TATA-box-binding protein (TBP) is an integral component of the transcription initiation complex required by all three eukaryotic RNA polymerases [30]. TBP contains a highly conserved core domain at the C-terminal region that mediates many of its transcriptionally relevant interactions in eukaryotes (Figure 2a). This C-t region has a symmetric structure with two well-conserved TBP domains that mediate binding to the targeted DNA [31]. On the contrary, the N-terminal tail of TBP is evolutionarily divergent and modulates the DNA-binding ability of its C-terminal part [32]. Interestingly, the N-terminal segment contains a low complexity region with a Q-rich motif (that varies in length from 25 to 42 residues in healthy individuals) that is thought to be involved in transcriptional activity regulation [33]. Detailed sequence analysis of TBP with PLAAC, a program aimed to identify PrLDs in protein sequences [34], revealed the presence of a 120-residues N-terminal segment with prion-like amino acid composition (Figure 2a and Figure 2b). According to COILS [9] and PARCOIL2 [10], two algorithms intended to identify sequences with a high CC propensity, the N-terminal tail of TBP also encodes a sequence of about 45 residues with a high propensity to form a CC that overlaps with the Q-rich and PrLD motifs (see Figure 2a and Figure 2c-d). This region comprises six predicted continuous heptad repeats (Figure 2a and Figure 2e) built mainly by Gln residues (78%).

Although the function of the PrLD domain from TBP remains unclear, it may likely play a role in regulating TBP function, since the N-terminal region where it resides can repress the ability of the TBP to bind the TATA box and at the same time can permit cooperative binding with other basal factors.

Detection of the TATA-BOX by TBP is an essential step for the formation of a transcription initiation complex. In this process, TBP binds to TATA-BOX sequences in two different steps. The first step requires the interaction of the inhibitory DNA-binding (IDB) surface from the C-terminal part of TBP to positively regulate the formation of the unstable DNA unbent complex. The second step involves a conformational switch that leads to forming a stable DNA bent complex [35]. The N-terminal region inhibits the formation of the bent complex and favours that of the unbent one, and conformational changes in this region are necessary to form the active assembly.

Recent cryo-EM structural studies have revealed many details concerning the early steps of TFIID assembly [36]. TBP interacts with up to 13 different TBP-associated factors (TAFs) to build up the TFIID complex, with six of them (TAF4, TAF5, TAF6, TAF9, TAF10, and TAF12) present in two copies. This large macromolecular complex of about 1.3 MDa is essential for recognizing core promoter sequences and recruiting the preinitiation complex (PIC) during basal transcription. Our previous analysis of TBP-associated factors (TAFs) revealed an overrepresentation of Q-rich sequences and overlapping CCs motifs [3]. This trend has been previously described by Fiumara and co-workers in yeast, who reported that many of the protein interactors of yeast functional prions contain CCs [7]. For instance, human TAF4 contains four glutamine-rich (Q-rich) domains involved in mediating interactions with transcriptional activators.

TBP has also been linked to the key pathological features of some neurodegenerative disorders, including neuronal intranuclear hyaline inclusion disease (NIH-ID) [37], spinocerebellar ataxia types 1, 2, 3 (SCA1, SCA2, SCA3) [38,39], dentatorubral-pallidoluysian atrophy (DRPLA) [38], Huntington’s disease (HD) [40], and Alzheimer’s disease (AD) [41]. In particular, expansion of the polyQ tract beyond 42 residues in TBP has been associated with the development of cerebellar ataxia (SCA17), an autosomal dominant and progressive neurodegenerative disease [42]. Pathological expansion in its polyQ domain has been proposed to induce neurodegeneration. This expansion caused TBP aggregation and precipitation within neurons [43,44], decreased its dimerization, altered its binding to other transcription factors. This behaviour is compatible with the presence of sequence stretches able to assemble into highly order amyloid-like structures. The emerging picture is that these so-called soft amyloid cores (SAC), are necessary for prion conversion in yeast as well as in human cells [3]. We identified a similar region in TBP that comprises residues 93-QQQQAVAAAAVQQSTSQQATQ-112 by using pWALTZ [45]. This sequence is located immediately after the CC motif and connects with the adjacent doublet of TBP domains (Figure 2a). The presence of this SAC might facilitate amyloid formation by decreasing the energy barrier for the conformation transition, with the adjacent CC region facilitating the subsequent intermolecular contacts, as demonstrated for MED15 [3].

In summary, TPB plays an essential role in transcription initiation, and the presence of PrLD in this protein might be important for this function, through its binding to DNA or by its interaction and regulation of/by other transcriptional players. The questions are if, and how, mutations/expansions at its Q-rich region might promote amyloid conversion and which would be the role of CCs in this putative transition. Answering them, should help to understand the contribution of the PrLD identified in TBP to disease onset and progression.

### cAMP-RESPONSE ELEMENT-BINDING PROTEIN (CBP)

**Figure 3.**
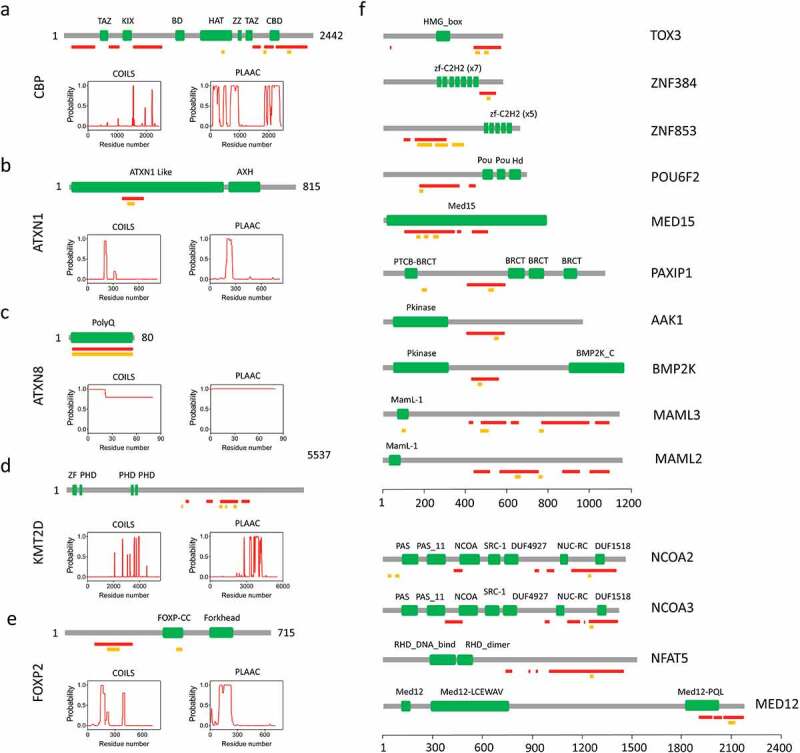
**Human proteins containing PrLDs and overlapping CC regions**. a-e) Linear representation of a) CBP, b) ATXN1, c) ATXN8, d) KMT2D, and e) FOXP2 showing the location of the Pfam domains (green) (3). The prion-like domains (red) based on PLAAC [34] predictions (0.8–1 score) and the CC regions (yellow) using COILS [9] predictor are indicated (0.8–1 score). In a-e), the COILS and PLAAC per-residue probability plots have been included. f) Linear representation of other human prion-like proteins with overlapping CCs. Note that the COILS score (> 0.3) has been considered for AAK1 and NFAT5

cAMP-response element-protein-(CREB) binding protein (CBP) is a transcriptional coactivator localized in the cell nucleus. CBP has a lysine acetyltransferase domain (HAT in Figure 3a) located in the central region of the protein and catalyzes the acetylation of target proteins [46]. CBP interacts with a variety of cell signalling proteins, especially those with established pro-survival effects in neurons, such as the CRE-binding protein (CREB) [47] and plays a crucial role in critical biological processes, such as embryogenesis, development, differentiation, and apoptosis [48]. Thanks to its HAT motif, CBP is endowed with histone acetyltransferase activity, promoting the acetylation of histones, which influences chromatin condensation and is a key mechanism in regulating transcription [46]. On the other hand, the N- and C-terminal domains of CBP can act as transactivation domains, and the protein also contains three potential α-helical motifs containing the sequence L*XX*LL (amino acids 68–78, 355–365, and 2067–2077) called NR boxes, to mediate interactions with nuclear receptors. The C-terminal NR box lies within the C-terminal low complexity region of CBP and interacts with other coactivators, including activator for thyroid hormone and retinoid receptors (ACTR) and the steroid receptor coactivator 1 (SRC-1) [49–51].

Detailed sequence analysis of CBP showed that this protein contains up to six discontinuous segments with prion-like features (Figure 3a). In fact, the C-terminal PrLDs identified in this protein overlap with two predicted CC motifs and with a tract of 18 consecutive Gln residues near its carboxy-terminal tail (residues 2199–2216). This polyQ motif has been involved in the sequestration of CBP to aggregates of several distinct polyQ proteins [52]. Indeed, CBP dysfunction has been identified in a number of neurological disorders, such as the Rubinstein–Taybi syndrome (RTS), AD, Amyotrophic Lateral Sclerosis (ALS) and polyQ-related diseases, in which CBP has been found in the inclusion bodies formed by polyglutamine-containing proteins in HD, DRPLA, SCA3, SCA7, and spinal and bulbar muscular atrophy (SBMA) [52–56]. CBP depletion from its normal location and sequestration into aggregates through interaction with expanded polyQ proteins has been suggested to be mediated by its C-terminal polyQ tract [52]. As a result, impaired CBP-mediated transcription and the lack of CBP-dependent acetylation are associated with increased cell death rates. It is tempting to propose that the C-terminal region of CBP and specifically its Q-rich, CC-containing, PrLD might be involved in a conformational switch, similar to the one described for MED15, which might facilitate polyQ-mediated interactions and CBP aggregation.

## ATAXIN-1 (ATXN1) AND ATAXIN-8 (ATXN8)

The spinocerebellar ataxias (SCAs) are a group of autosomal dominantly inherited progressive disorders associated with cerebellar degeneration and progressive ataxia [57]. Almost thirty different genetic causes of SCAs have been reported so far and are numbered chronologically in order of discovery [57]. Most of them have shown to progress with classic cerebellar signs [57]. However, many variants display disabling non-cerebellar features such as brainstem dysfunction, eye movement abnormalities, and visual loss [57].

Ataxin-1 (ATXN1) is an RNA binding protein with a role in transcriptional regulation. ATXN1 contains an RNA-binding motif (residues 541–767) comprising a globular AXH domain (residues 568–689) involved in ATXN1 interactions. Predictions using PLAAC and COILS suggested that ATXN1 encodes prionic traits at the N-terminal region, with five CC heptad repeats overlapping with the detected PrLD (Figure 3b).

Mutations in the ataxin-1 gene have been associated with the development of spinocerebellar ataxia type 1 (SCA type 1). This protein is located in the nucleus and interacts with RBM17 (a splicing factor) and several transcription regulators, including SMRT, HDAC3 an Capicua [58–60]. Wild-type ATXN1 has a length of 816 residues. The presence of a trinucleotide repeat disorder caused by the expansion of the CAG repeat in the N-terminal region of ATXN1 leads to an expanded polyQ tract and disease development. This polyQ tract (residues 197–225) is located at the N-terminal region of the protein, mapping at the predicted PrLD and CC motifs. This expansion in ATXN1 favours its binding to the transcriptional repressor protein Capicua homolog via the RNA- binding protein and spliceosome component RBP7. This aberrant binding leads to a disruption in gene expression and splicing events in the affected neurons [57]. ATXN1 shuttles between the nucleus and cytoplasm. However, ATXN1 dynamics are altered by the Gln expansion and failure in nuclear export upon polyQ expansion dramatically reduces the retro-transport into the cytoplasm [61]. Regardless of the polyQ expansion, subtle increases in wild type ATXN1 levels could also lead to SCA1 hallmarks [62], whereas decreasing ATXN1 expression helps to reduce ATXN1’s accumulation and mitigates cerebellar SCA1 pathogenesis [63–66]. Conversely, it also has been shown that loss of the protein increases the levels of BACE1 and Aβ pathology, indicating that ATXN1 levels is a potential factor for AD development [67]. Co-regulation by microRNAs (miRNAs) together with the RNA-binding protein PUMILIO1 (PUM1) are shown to fine-tune posttranscriptionally ATXN1 mRNA levels [62,63,68]. Posttranslational modifications such as phosphorylation, transglutamination, ubiquitination, and sumoylation are also required for the ATXN1 function [60]. Furthermore, the N-terminal α-helical CC domain in the PrLD of MED15 has been shown to efficiently promote spontaneous ATXN1 aggregation in vitro, implying that CC regions in modifiers are essential to promote aggregation and toxicity effects on polyQ diseases [69].

Much less is known about ATXN8. Expansions in this protein have been linked to spinocerebellar ataxia type 8 (SCA8) in humans. These polyQ expansions showed a reduced penetrance compared to the expansions observed for other SCA-associated disease genes [57]. A difference with ATXN1 is that the expansion on CAG repeats in ATXN8 leads to a nearly pure polyQ protein. As shown in Figure 3c and according to PLAAC and COILS predictions, almost all the 80-residues of ATXN8 map to a PrLD with the potential to acquire a CC fold. It remains unclear how the structural features of proteins bearing long polyQ stretches are translated with toxic phenotypes. This might depend on the unique capacity of these proteins to target other cellular proteins through CC-to-CC interactions. The concept of CC-mediated aggregation also provides a plausible mechanism for polyQ-protein deposition in the brain.

## LYSINE METHYLTRANSFERASE 2D (KMT2D)

The *KMT2D* gene (also called MLL4, ALR, or MLL2) is a member of the SET family of histone methyltransferase enzymes. *KMT2D* gene in human maps to 12q13.12, including over 19 kb pairs in length and contains up to 56 exons, encoding a large polypeptide with 5537 amino acids. The transcript of this gene has a molecular weight of 593 kDa and catalyzes the methylation (either mono-, di-, and trimethylation) on lysine 4 (K4) of the histone H3 protein (H3K4). This is an important histone modification that controls transcriptionally active promoters and enhancers [70], increasing the transcription of the gene packaged around the histones [71].

KMT2D contains a cluster of conserved domains at the C-terminal end, comprising a PHD (plant homeodomain)-zinc-finger like domain, two phenylalanine, and tyrosine (FY)-rich motifs (F/Y-rich N-terminus (FYRN) and F/Y-rich C-terminus (FYRC)) and a catalytic SET domain (SET: Su(var)3–9, Enhancer-of-zeste (E(z)), and Trithorax) that are needed for its enzymatic function [72]. KMT2D is a major regulator of cell-type-specific gene expression in cell differentiation during tissue development and embryogenesis [73,74]. In addition, KMT2D has also been related to tumour suppression and immune signalling [74–76].

In humans, KMT2D contains a high predicted disorder content of (55%), a common characteristic of nuclear proteins involved in transcription and chromatin organization, with a series of polyQ tracts at the C terminus end of the highly conserved central regions. These regions are part of a large low complexity segment, consisting of four discontinuous regions with predicted prion-like features (Figure 3d), with the largest prion-like region containing a set of three consecutive CC motifs that overlap with the polyQ repeats.

Most *KMT2D* mutations are assumed to cause truncated proteins that do not perform the function properly due to loss of the catalytic SET domain [72]. Mutated KMT2D has been linked to developmental disorders including Kabuki syndrome [77], congenital heart disease [78], and multiple cancer types such as medulloblastoma, lymphoma, hepatocellular carcinoma, gastric cancer, breast cancer and prostate cancer [73,74]. This pleiotropic effect results from the fact that its pathogenic variants cause the interruption of histone methylation and abnormal enhancer regulation, leading to changes in transcription, thus affecting normal growth and development [74]. The exon 39 (3581–4510 aa) constitutes a mutational hotspot [79] and, interestingly enough, it contains the most extended prion-like domain, with missense disease-associated mutations clustering at the predicted CC and Q-rich motifs comprising residues 3897–3975 [70]. Whether such mutations predispose to disease by disrupting normal protein-protein interactions, mediated by KMT2D during chromatin remodelling, or promoting a conformational transition towards an aggregated state remains to be elucidated.

## FORKHEAD BOX PROTEIN P2 (FOXP2)

FOXP2 is a multifunctional transcription factor expressed in various brain regions and peripheral organs during embryonic development and adulthood [80,81]. In humans, FOXP2 has 715 residues (80kDa) and is one of the most highly conserved proteins in vertebrate genomes [82]. It has been shown to impact gene regulation in multiple aspects of neuronal development [83–85].Indeed, Forkhead-box protein P2 (FOXP2) was one of the first genes to be linked to human language disorder, characterized by a broader cognitive dysfunction and primary motor impairment. Thus, this protein is essential for the normal development of speech and language [86–88]. Human oncogenesis has also been linked to dysregulated FOXP2 function [89].

FOXP2 contains different sequence motifs, including a single C2H2 zinc finger (C2H2-ZF) motif and a leucine zipper (LZ) motif that mediates FOXP2 dimerization through CC formation. This domain has a critical role in DNA binding and promotes heterotypic and homotypic protein interactions with FOXP1/2/4 family members [90,91]. In addition, FOXP2 has a strongly conserved forkhead DNA-binding domain (FHD, 100-aa or ‘winged-helix”) at the C-terminus that can form a domain-swapped dimer, and two nuclear localization signals (NLS) [92,93]. As shown in Figure 3e, a long and disordered predicted PrLD lies near the N-terminus in human FOXP2. It overlaps with a glutamine-rich region, that contains a long perfect polyQ repeat made of up to 40 CAG/CCG repeats (p.Q152-Q191) and a shorter imperfect repeat (p.Q200-Q230) adjacent to it, with both Gln-rich regions displaying a high propensity to form CCs, according to COILS. Thus, FOXP2 shares all the sequential features shown to promote a CC to amyloid transition in the case of MED15 and it can be considered a potential functional human prion-like protein.

## OTHER PREDICTED PRION-LIKE HUMAN PROTEINS WITH COILED-COILS

As previously mentioned, a total of 20 human prion-like domains (PrLDs) containing high CC propensity sequences that, in the majority of cases, overlapped with polyQ stretches were identified in the human proteome [3]. These predicted prion-like proteins work in the regulation of transcription, and they comprise key transcription coactivators, including MED12, MED15, MAML3 and MAML2, CBP, protein kinase and ATP binding activities like BMP2K, AAK1 or DNA-binding transcription factors such as TBP, NFAT5, NCOA3, NCOA2 and FOXP2 (Figure 3f). For most of them, it is unknown whether they can access a prionic state and, in this case, if α-helical CC within PrLDs would be self-sufficient mediators of functional prions aggregation or just intermediates or facilitators in the β-sheet formation process. In any case, we foresee an increasing interest in studying the verisimilitude of these transitions, provided the functional relevance of this prion-like proteins subset.

## CONCLUSIONS

Recent observations suggest that the coincidence of low complexity regions and CC-forming sequences in the PrLDs of prion-like proteins is a frequent feature that might be critical for their function. In humans, these proteins are key functional mediators in transcription and are associated with divergent disorders such as neurodegenerative diseases or cancer. Although only a reduced set of functional prions have been deeply characterized, compared to the vast number of pathologic amyloid proteins, some specific traits can already be delineated. A feature common to almost all prion-like proteins is the spatial separation of the active globular and prionic domains. The prionic traits are concentrated in low complexity regions, which, as described here, might display a high propensity to form CCs and mediate protein-protein interactions, either directly or through a folding-upon-binding mechanism. Many human prion-like proteins interact with DNA and/or RNA, a property mediated by their globular domains, and that for some of them is associated with their liquid-liquid phase separation and the formation of membraneless compartments. Although it is assumed that pathological conditions result from the inherent toxicity acquired by the aggregates of these proteins, our results with MED15 suggest an alternative explanation in which cytotoxicity is associated with the sequestration of the protein in the wrong compartment, its inactivation upon clustering with a consequent loss of crucial protein-protein interactions or conversely the establishment of undesired intermolecular interactions with dysregulate signalling pathways. The CCs would be important players in these processes, and indeed a significant number of pathological mutations map into these domains, either decreasing or increasing their helical propensity.

Despite progress in the last decades in the field of prions and amyloids, many questions remain to be answered. For example, we still need to address how human prion-like proteins participate in transcriptional mechanisms under specific environmental conditions and how dynamic are the stimuli that trigger their amyloid conversion *in vivo*. Another crucial question is how the conformational changes in PrLDs occur and how they lead to amyloid formation. The structural flexibility of low-complexity regions and the CC-mediated transition model provides a plausible mechanism to explain the functional to pathogenic conversions of at least a fraction of these polypeptides. Providing molecular evidence for such conformational transition in the proteins discussed in this review might uncover an additional layer of transcription regulation and help to understand why these proteins are indefectibly associated with human disease.
